# Oxidative Stress in Biotoxin-Induced Liver Injury: From ROS Generation to Cell Death and Therapeutic Intervention

**DOI:** 10.3390/toxics14070625

**Published:** 2026-07-17

**Authors:** Ming-Ye Hu, Xiang-Yu Hou, Xiao Huang, Xue-Lu Yu, Fang Liu, Ji-Shun Yang

**Affiliations:** 1 College of Pharmacy, Bengbu Medical University, Bengbu 233030, China; 18155665702@163.com; 2 Naval Medical Center of PLA, Naval Medical University, Shanghai 200433, China; hxy15846783301a@163.com (X.-Y.H.); 18916873358@163.com (X.H.); selinayxl@163.com (X.-L.Y.)

**Keywords:** biotoxins, hepatic injury, oxidative stress, mechanisms

## Abstract

Exposure to diverse naturally occurring biotoxins—originating from fungal, plant, and algal sources—poses a severe and escalating threat to global public health. The liver, due to its pivotal role in toxin metabolism, faces an increased risk of injury from toxin accumulation. Although extensive research has been conducted on toxin-induced hepatic injury, the heterogeneity of oxidative stress mechanisms across different injury phenotypes (such as necrosis, steatosis, and fibrosis) remains poorly understood. This review systematically addresses this gap by establishing oxidative stress as the central, convergent mechanism underlying biotoxin-induced liver injury. By critically comparing representative toxins, we examine the role of oxidative stress in various biotoxin-induced hepatic injury phenotypes and their underlying molecular mechanisms. Furthermore, we discuss the specific involvement of interconnected signaling cascades—specifically the Nrf2, NF-κB and MAPK pathways—in orchestrating different modes of cell death (including apoptosis, ferroptosis, and necroptosis), and summarize antagonistic strategies for biotoxin-induced hepatic injury from an oxidative stress perspective, highlighting their translational potential and providing a robust rationale for developing broad-spectrum, redox-targeted therapies.

## 1. Introduction

In nature, various organisms synthesize a wide array of toxic secondary metabolites collectively known as biotoxins, which are primarily categorized by their origin into mycotoxins, phytotoxins, and algal/cyanobacterial toxins. Liver diseases represent a massive global health burden, affecting millions of people worldwide and constituting a critical public health challenge. For instance, aflatoxins exposure is accountable for 4.6% to 28.2% of global hepatocellular carcinoma cases annually [1], while pyrrolizidine alkaloids (PAs) remain the leading cause of hepatic sinusoidal obstruction syndrome (HSOS) in specific regions like China, accounting for 50% to 89% of reported cases [2]. Furthermore, aquatic toxins such as microcystins (MCs) pose severe threats; acute exposure has historically caused 76 fatalities in a single hemodialysis water contamination outbreak [3], and chronic childhood exposure (with estimated daily intake up to 2.03 μg, far exceeding the WHO recommended limits) is linked to elevated liver enzymes and the progression of metabolic liver diseases [4]. Human exposure to these toxins occurs via contaminated food, water sources, occupational contact, or environmental events, leading to diverse adverse outcomes ranging from acute poisoning to chronic organ dysfunction [5]. The liver serves as a central hub for biotransformation and metabolic regulation, undertaking multiple physiological functions through its unique architecture. Anatomically, its dual blood supply system predisposes the liver to accumulate toxins from the gastrointestinal tract, acting as a metabolic nexus for toxins [6], thereby bearing heightened risks of toxin exposure and injury.

Although biologically induced hepatic injury exhibits highly heterogeneous pathological manifestations—including cirrhosis, acute and chronic hepatic necrosis [7], fibrosis [8], and even tumorigenesis [9]—extensive evidence indicates that oxidative stress plays a pivotal role in the initiation and progression of these hepatic pathologies. Oxidative stress occurs when reactive oxygen species (ROS) production (superoxide anion, hydrogen peroxide and hydroxyl radical) exceeds the clearance capacity of antioxidant defenses, leading to downstream consequences such as lipid peroxidation and DNA damage [10]. It arises through multiple toxin-specific pathways involving excessive ROS generation, imbalance of antioxidant defenses, mitochondrial dysfunction, inflammatory responses, and activation of cell death pathways. Compounding this vulnerability, the liver is particularly susceptible to oxidative stress due to its high metabolic activity and abundant cytochrome P450 (CYP450) enzyme systems [11]. Excessive oxidative stress ultimately triggers multiple regulated and unregulated cell death pathways, thereby influencing the clinical outcome of diseases.

Upon exposure, toxins induce primary ROS generation, causing direct cytotoxicity. It should be noted that ROS are not merely cytotoxic mediators but subsequently also serve as signaling molecules exploited by numerous biotoxins to reprogram host responses. Certain toxins establish redox environments conducive to their persistence or pathogenic effects by modulating ROS production [12]. In response, hepatocytes and non-parenchymal liver cells initiate a series of adaptive or pathological responses. These include suppressing the activation of antioxidant defense pathways, notably the Kelch-like ECH-associated protein 1-Nuclear factor erythroid 2-related factor 2/Antioxidant Response Element (Keap1-Nrf2/ARE) pathway [13], upregulating inflammatory signaling pathways (such as NF-κB and MAPK) [14], and activating cell death programs (such as apoptosis, necroptosis and ferroptosis) that drive disease progression [15]. Recent studies indicate that organelle interactions also trigger excessive ROS production [16]. Beyond direct cellular damage, accumulating evidence suggests that the gut–liver axis plays a key role in the hepatotoxicity of biotoxins. Toxins can directly or indirectly disrupt the intestinal barrier by causing gut microbiota dysbiosis, leading to the translocation of endogenous microbial products (such as lipopolysaccharide, LPS) into the portal vein. These products then act synergistically with ingested exogenous toxins to exacerbate hepatic oxidative stress and inflammatory responses [17]. Understanding these interconnected redox-dependent mechanisms is crucial for rationally developing diagnostic biomarkers and therapeutic interventions.

Despite the existence of numerous excellent reviews that have deeply explored the hepatotoxic mechanisms of specific categories of biotoxins (aflatoxin B1, AFB1) [18], there remains a notable lack of systematic comparisons across biotoxins from different sources (fungi, plants, bacteria/cyanobacteria) regarding how they drive diverse liver injuries through a common oxidative stress hub. Previous reviews frequently focus on isolated molecular pathways, leaving a conceptual gap regarding how diverse biological agents act collectively. To address this, the present review provides a unified analytical framework across toxin categories. We not only systematically elucidate the specificities and common pathways of oxidative stress induction by representative biotoxins from each category, but also emphasize a comparative analysis of the interactions among core downstream signaling networks (Nrf2, NF-κB, MAPK) of oxidative stress and their regulation of different cell death modalities (apoptosis, ferroptosis, necroptosis). Additionally, we incorporated discussions on emerging frontier concepts such as the gut–liver axis and nuclear receptor interference [19], linking hepatoprotective strategies to specific toxin models. The value of this paper lies in its comparative toxicological perspective, aiming to provide theoretical references for understanding the common oxidative stress patterns underlying heterogeneous liver injury phenotypes, as well as for developing broad-spectrum or precise intervention strategies. To achieve this, we deliberately selected AFB1 and ochratoxin A (OTA), PAs and ricin, and lipopolysaccharides and MCs as our focal models. These specific agents were chosen because they represent the most epidemiologically significant and mechanistically diverse toxins across three fundamentally distinct biological kingdoms [1,2,4]. This specific cross-category selection provides a robust analytical framework, allowing us to scientifically establish our central hypothesis: that different biotoxins, utilizing different routes of entry, ultimately converge on a shared pathway of oxidative stress, leading to similar underlying mechanisms of liver damage.

## 2. Literature Search Strategy

### 2.1. Literature Selection

This literature review primarily screened relevant studies published in authoritative academic journals between 2001 and 2026. The primary databases searched included PubMed and Web of Science. Inclusion criteria prioritized studies directly investigating how diverse biotoxins induce hepatic cell injury, apoptosis, or fibrosis via oxidative stress pathways. Systematic retrieval using keywords such as “biotoxins”, “oxidative stress”, “hepatic injury”, “apoptosis”, and their combinations yielded 106 representative high-quality publications, collectively outlining the latest advances in this research domain.

### 2.2. Inclusion Criteria

To ensure analytical depth and reliability, we prioritized original research articles involving in vivo animal models (mice or rats) and in vitro experiments (the HepG2 cell line) concerning biotoxin exposure. The selected studies were required to focus primarily on the liver or hepatocytes and specifically investigate biological toxins such as AFB1, OTA, PAs, ricin, LPS, or MCs. Furthermore, research had to explicitly address oxidative stress-related mechanisms by measuring parameters like ROS levels, antioxidant enzyme (Superoxide Dismutase, SOD) activity, lipid peroxidation products (Malondialdehyde, MDA), or investigating related signaling pathways (including Nrf2). Studies that lacked a primary focus on hepatotoxicity or failed to detail mechanistic pathways were excluded. Finally, the eligible literature was restricted to peer-reviewed original research and reviews published between 2001 and 2026 to capture the latest advances in the field.

## 3. Hepatotoxicity Characteristics of Biotoxins and the Central Role of Oxidative Stress

Biotoxin-induced hepatic injury represents one of the most prevalent forms of toxic hepatopathy. Despite the mechanistic complexity underlying hepatic injury caused by diverse biotoxins—such as PAs from plants, aflatoxins produced by molds, and MCs in aquatic environments—oxidative stress emerges as a ubiquitous pathogenic driver. This section outlines the hepatic pathological phenotypes and primary direct toxic mechanisms triggered by several representative biotoxins (Table 1).

### 3.1. Fungal Toxins (AFB1 and OTA)

Aflatoxins are secondary metabolites produced by *Aspergillus* fungi in moldy grains, legumes, nuts and other foods. Approximately 20 derivatives exist, with AFB1 being the most widely distributed and potent variety in nature [39]. It is classified as a Group 1 carcinogen by the International Agency for Research on Cancer [40]. Human ingestion of high doses of aflatoxins induces rapid-onset acute hepatotoxicity, manifesting clinically as a progression from fever, vomiting, anorexia, and jaundice to ascites, lower limb edema, and high mortality [41]. Animal models have shown that pathological changes in the liver of pregnant rats exposed to aflatoxin include acute lesions such as hepatic parenchymal cell necrosis, bile duct epithelial cell proliferation, fatty infiltration of the liver, and hepatic hemorrhage [20]. Data also suggest that chronic aflatoxin exposure can induce hepatocellular carcinoma [42]. AFB1 undergoes metabolism in vivo via hepatic CYP450 enzymes to form AFB1-8,9-epoxide, which covalently binds to DNA, RNA, and proteins, initiating mutagenic and cytotoxic cascades [21]. CYP450-mediated AFB1 metabolism concurrently generates ROS, propelling lipid peroxidation, mitochondrial dysfunction, and glutathione (GSH) depletion, which collectively amplify oxidative damage and apoptotic signaling [22].

Ochratoxins are secondary metabolites produced by *Aspergillus* and *Penicillium* species, widely contaminating agricultural products and animal feed such as cereals, grapes, and nuts. They constitute a class of mycotoxins with potent nephrotoxic and carcinogenic properties [43]. Ochratoxins are primarily classified into OTA, Ochratoxin B (OTB), and Ochratoxin C (OTC) based on structural differences. Among these, OTA exhibits the widest natural distribution and the most significant toxicological relevance, classified by the International Agency for Research on Cancer as a Group 2B probable carcinogen [44]. Ochratoxins primarily affect the kidneys and liver in rats. Despite predominant nephrotoxicity, the literature demonstrates that 4 weeks after administration of 250 µg/kg OTA, rat hepatocytes exhibit granular or vacuolar degeneration and necrosis, dilation of sinusoids and central veins, bile duct hyperplasia, and mild fibrous tissue proliferation [24]. OTA increases oxidative stress levels in liver and kidney tissues in a dose-dependent manner, including lipid peroxidation [25]. OTA disrupts mitochondrial dynamics and structure in hepatocytes and nephrocytes, leading to mitochondrial homeostasis dysfunction. Mechanistically, OTA may contribute to oxidative stress development by impairing key antioxidant enzymes, such as catalase and GSH peroxidase, thereby elevating intracellular H_2_O_2_ levels [26].

### 3.2. Plant Toxins (PAs and Ricin)

PAs constitute a widely distributed class of natural toxins in the plant kingdom, characterized by a bicyclic nitrogen-containing structure [45]. These compounds are primarily found in plants of the Boraginaceae, Asteraceae, and Fabaceae families, such as dog’s-tooth violet and Korean knotweed. The unsaturated 1,2-double bond and hydroxyl substitution are key structural features conferring toxicity [46]. It is estimated that approximately half of the 660 characterized PAs and PA N-oxides exhibit cytotoxicity, genotoxicity, and carcinogenicity [27]. PA poisoning causes severe acute hepatic injury, commonly termed HSOS, with clinical manifestations including abdominal distension, hepatic pain, ascites, jaundice, and hepatomegaly. Notably, in Western settings, HSOS is frequently associated with myeloablative conditioning prior to hematopoietic stem cell transplantation (HSCT) [28]. While chemotherapeutic agents like cyclophosphamide are commonly recognized as inducing HSOS through mechanisms involving lipid peroxidation and protein oxidation in the liver [47], PA-induced hepatotoxicity follows a distinct pathway. Specifically, PAs are first metabolized by CYP450 to produce dehydropyrrolizidine alkaloids (DHPAs) and/or dehydroretronecine (DHR), which subsequently deplete hepatic GSH and induce mitochondrial dysfunction, leading to excessive ROS production [29]. High levels of ROS can activate Nrf2-mediated ARE gene transcription, induce Nrf2 nuclear translocation, and initiate the endogenous antioxidant system. However, this compensatory effect is limited and cannot completely reverse the liver injury caused by ROS accumulation [30].

Ricin is a highly toxic plant protein extracted from castor seeds, consisting of two polypeptide chains (chain A and chain B) linked by a disulfide bond to form a heterodimer, and belongs to type II ribosome-inactivating proteins [31]. Due to its high toxicity and easy availability, ricin has been listed as an important biological terror agent and is strictly controlled by the international community [48]. The toxic mechanism of ricin is also inseparable from oxidative stress. Ruijiao Lin [32] established an SD model of ricin poisoning by intraperitoneal injection of 23 µg/kg of toxin, indicating that ricin can induce acute liver injury in rats, and the mechanism may be related to mitochondrial damage caused by decreased levels of reduced GSH and protein levels of GSH peroxidase 4 (GPX4) and solute carrier family 7 member 11, as well as increased levels of iron, MDA, and reactive oxygen species in the liver.

### 3.3. Bacterial Toxins (LPS and MCs)

LPS constitutes the primary component of the outer membrane in Gram-negative bacteria, accounting for approximately 75% of the outer membrane’s mass [49].

It is one of the most potent endotoxins known to date. LPS comprises three structural domains: O-antigen, core polysaccharide, and Lipid A. Its detrimental effects manifest primarily when it enters the bloodstream in large quantities, triggering a systemic inflammatory response. In severe cases, this can lead to sepsis, septic shock, or even multiple organ failure [50]. LPS has been demonstrated to cause both acute and chronic hepatic injury. A mouse model of endotoxemia is typically established via intraperitoneal LPS injection to simulate sepsis-associated acute hepatic injury. As LPS levels progressively exceed hepatic tolerance thresholds, it stimulates hepatic macrophages to release ROS, activating the TLR4/NF-κB pathway and amplifying the inflammatory cascade, thereby inducing hepatic injury. ROS not only directly attacks hepatocytes and induces oxidative damage, but also acts as an upstream signaling molecule to activate the TLR4/NF-κB inflammatory pathway, promoting massive release of inflammatory factors, forming a vicious cycle of inflammation and oxidative stress, and ultimately aggravating liver tissue lesions [33].

MCs are cyclic heptapeptide secondary metabolites produced by freshwater cyanobacteria (such as *Microcystis*, *Anabaena* and *Aphanizomenon*) and belong to the class of hepatotoxins [51]. Due to their high toxicity, environmental persistence, and bioaccumulation, MCs are designated by the World Health Organization as critical monitoring parameters for drinking water safety. The hepatotoxicity of MCs is closely linked to their specific inhibition of protein phosphatase 1 (PP1)/phosphatase 2a (PP2A) [35]. Exposure can lead to cytoskeletal disassembly, ROS bursts, depletion of GSH, disruption of redox homeostasis, and even DNA damage [36]. Histopathological studies indicate that MCs induce hepatic hemorrhage, necrosis, steatosis, and inflammatory cell infiltration [37]. Furthermore, as the primary target organ for MCs, the liver has been implicated in the development of hepatocellular carcinoma, non-alcoholic fatty liver disease, and liver fibrosis, with significant associations demonstrated between MC exposure and these conditions [35].

## 4. Toxin-Mediated ROS Generation Mechanism

**Figure 1:**
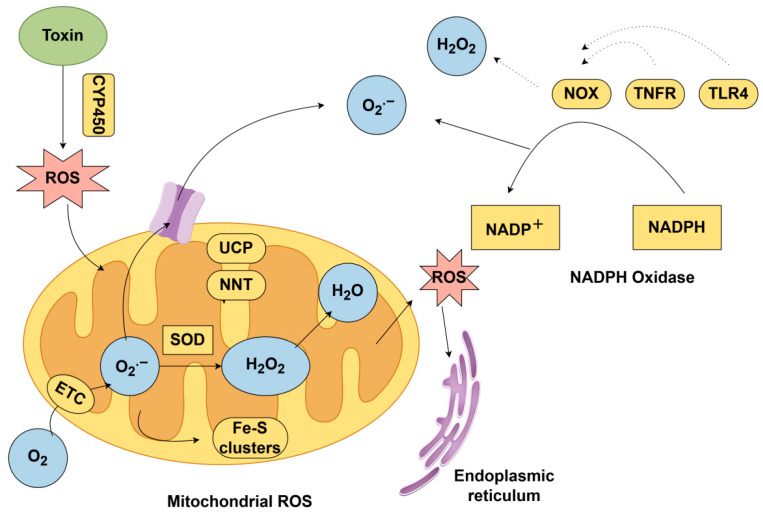
Mechanism of ROS generation mediated by toxins. The mechanism of toxin−mediated ROS generation involves multiple interconnected pathways: (1) Primary ROS generation: Exogenous toxins enter hepatocytes and can undergo metabolism via the CYP450 enzyme system, producing primary ROS as byproducts during the catalytic redox cycle [52]. Alternatively, toxins directly target mitochondria, disrupting the ETC and causing electron leakage, which leads to the direct accumulation of O_2_^.−^ [56,57]. (2) Secondary ROS amplification: The initial oxidative stress acts as a signaling mechanism to activate membrane receptors, such as TLR4 and TNFR [34]. This activation triggers inflammatory signaling cascades that stimulate NOX located on the membrane or endoplasmic reticulum. NOX utilizes NADPH to generate massive amounts of secondary ROS [54,58]. (3) Vicious cycle and defense failure: Mitochondrial ROS can cross−talk with NOX, forming a positive feedback loop [59]. Although endogenous antioxidant systems, such as SOD, attempt to neutralize O_2_^.−^ into H_2_O_2_ and water [10], this continuous cyclical generation of ROS ultimately overwhelms the hepatic antioxidant defense capacity, leading to severe cellular damage. Abbreviations: CYP450, Cytochrome P450; ETC, Electron transport chain; Fe−S clusters, Iron-sulfur clusters; H_2_O_2_, Hydrogen peroxide; NADP^+^/NADPH, Nicotinamide adenine dinucleotide phosphate; NNT, Nicotinamide nucleotide transhydrogenase; NOX, NADPH oxidase; O_2_^.−^, Superoxide anion; ROS, Reactive oxygen species; SOD, Superoxide dismutase; TLR4, Toll-like receptor 4; TNFR, Tumor necrosis factor receptor; UCP, Uncoupling protein.

ROS are primarily generated during cellular metabolic processes: the CYP450 enzyme system produces ROS during the redox metabolism of exogenous toxins [52]; concurrently, electron leakage within the mitochondrial electron transport chain (ETC) during energy production generates superoxide anions, particularly exacerbated by complex dysfunction [53]; Furthermore, the NADPH oxidase family can be activated by inflammatory or stress signals to generate ROS [54]. Recent studies indicate that organelle interactions also promote ROS production [55]. These pathways mutually reinforce each other, forming a vicious cycle that collectively leads to the outbreak of oxidative stress (Figure 1). In brief, oxidative stress is a central link in toxin-induced liver injury, and the massive accumulation of ROS is the most prominent feature and effect of this pathological process.

### 4.1. CYP450-Mediated Toxin Activation

The CYP450 family represents the most significant group of drug-metabolizing enzymes in the human body, responsible for the metabolism of endogenous and exogenous compounds [60]. CYP450 constitutes the largest enzyme family involved in NADPH- and/or O_2_-dependent hydroxylation reactions across all domains of life. In plants and animals, CYP450 plays a central role in the detoxification of xenobiotics [61]. Paradoxically, however, they may also bioactivate toxins into reactive intermediates that induce oxidative stress. This dual role depends on CYP450-mediated redox cycling. CYP450 can generate oxidative stress and promote disease progression by increasing ROS and altering redox balance through their catalytic cycles. Conversely, active intermediates or products formed from certain substrates after CYP450 modification may also contribute to disease development. Specifically, the metabolism of AFB1 in hepatocytes exemplifies this phenomenon. AFB1 undergoes CYP450-mediated metabolism to generate the highly reactive AFB1-8,9-epoxide [23]. This intermediate forms adducts with DNA, RNA, and proteins, inducing mutations and cellular damage [40]. During metabolism, the catalytic cycle of CYP450 enzymes is accompanied by ROS generation [62], which further induces lipid peroxidation, mitochondrial dysfunction, and GSH depletion, thereby amplifying oxidative stress responses. This mechanism not only underlies the potential toxicity of toxins but also reveals the dual role of enzyme-mediated metabolism in detoxification and toxicity potentiation within toxicological responses.

### 4.2. ETC Dysfunction

Mitochondria serve as both primary sources and targets of ROS. Under normal conditions, the ETC transmits electrons to oxygen molecules via redox reactions, generating water and coupling ATP synthesis. Minor electron leakage produces ROS, which are cleared by antioxidant enzymes [56]. However, Microcystin-LR (MC-LR) disrupts Complex I and III function, causing electron accumulation and leakage that generates superoxide anions. Concurrently, elevated membrane potential promotes reverse electron transfer to O_2_, exacerbating ROS production [57]. MC-LR reduces mitochondrial membrane potential by inducing ROS bursts, activates apoptotic proteins such as Bcl-2-associated X protein/Caspase-3, triggers the mitochondrial apoptosis pathway, and contributes to the progression of liver disease and various other conditions including metabolic disorders and cardiovascular diseases.

### 4.3. NADPH Oxidase (NOX) Activation

The NOX family represents one of the key enzymes for intracellular ROS production. Its activation significantly promotes oxidative stress, participating in diverse pathophysiological processes [58]. NOX can be activated by toxins via the TLR4/NF-κB pathway [34]. NOX-derived ROS can damage ETC complexes, whilst mitochondrial ROS induce feedback activation of NOX via the NLRP3 inflammasome, forming a vicious cycle that exacerbates oxidative stress [59].

## 5. Core Regulatory Network of Oxidative Stress and Cell Death Modes

The core regulatory network of oxidative stress is significantly activated under toxin exposure conditions. Its response mechanisms involve the synergistic and antagonistic interactions of key pathways including the Keap1-Nrf2/ARE [63], NF-κB [64], and MAPK pathways [65], collectively determining cellular survival, inflammatory responses, and ultimate fate.

The action of microcystin MC-LR can induce a rapid increase in intracellular ROS levels, and as a core second messenger, it simultaneously regulates multiple signaling pathways. On one hand, ROS modify cysteine residues in the Keap1 protein, promoting Nrf2 dissociation from the complex and nuclear translocation. By binding to antioxidant response elements (ARE), this initiates expression of phase II biotransformation enzymes (such as SOD, GSH synthase and NQO1) and antioxidant genes, enhancing cellular detoxification capacity and mitigating oxidative damage [66]. Notably, Nrf2 also indirectly blocks NF-κB nuclear translocation by inhibiting IκBα degradation, thereby extending beyond antioxidant effects to mediate anti-inflammatory mechanisms [67].

Conversely, ROS may also activate the IKK complex, leading to phosphorylation and degradation of IκBα. This releases NF-κB dimers p65/p50 into the nucleus, initiating transcription of key pro-inflammatory mediators such as TNF-α, IL-6, and COX-2 [68]. These factors further mediate multiple pathogenic pathways of hepatic injury: TNF-α induces mitochondrial apoptosis by activating the caspase-8/Bid pathway [69]; IL-6 promotes hepatic stellate cell activation and fibrosis [70]; and COX-2-derived PGE2 amplifies inflammatory signals, forming a positive feedback loop [71].

Concurrently, toxins such as MC-LR activate MAPK family members (JNK, ERK, p38) via ROS, participating in the fine-tuned regulation of apoptosis, inflammation, and proliferation. Activation of the JNK pathway further promotes oxidative stress and apoptosis; p38 enhances inflammatory cytokine production; while ERK signaling modulates the balance between survival and death in different contexts [72]. Crucially, during toxin stress, extensive crosstalk exists between MAPK and NF-κB pathways. For instance, JNK modulates NF-κB activity, while p38 participates in IL-6 expression regulation, forming a synergistic signaling network that amplifies injury [73]. This interactive signaling network further mediates and regulates various modes of cell death, driving the continuous progression of toxic injury.

### 5.1. Mitochondria-Dependent Apoptosis

Mitochondria-dependent apoptosis constitutes the core mechanism by which cells initiate programmed cell death under oxidative stress conditions [74]. Various toxins, including OTA [25] and MC-LR [57], notably trigger this pathway to induce hepatotoxicity. Under normal physiological conditions, mitochondria generate moderate levels of ROS via the respiratory chain, participating in cellular signaling and other physiological processes [75]. However, in mitochondria-mediated apoptosis, the B cell lymphoma 2 (Bcl-2) protein, typically located on the outer mitochondrial membrane, can block the pore formed by Bax, thereby preventing the release of cytochrome C and regulating apoptosis [76].

### 5.2. Ferroptosis

Ferroptosis is a newly discovered ROS-mediated non-apoptotic cell death characterized by the accumulation of lipid peroxides due to iron overload, leading to metabolic disruption [77]. Research indicates that ferroptosis plays distinct roles across various hepatic pathologies, potentially contributing to the progression of diverse inflammatory liver diseases. Plant toxins, such as ricin, specifically exploit this vulnerability by exacerbating iron accumulation and lipid peroxidation [32]. The Xc^−^ system promotes GSH synthesis, which serves as a cofactor for GPX4, the primary endogenous mechanism inhibiting lipid peroxidation. Toxins inhibit the Xc^−^ system or GPX4, blocking GSH synthesis and lipid peroxidation repair. This leads to acyl-CoA synthetase long-chain family member 4 (ACSL4)-mediated polyunsaturated fatty acid (PUFA) peroxidation, triggering cell membrane disruption [78].

### 5.3. Necroptosis

Necroptosis, also known as programmed necrosis, constitutes a regulated cell death pathway that morphologically resembles necrosis. Its defining characteristics include cell membrane rupture, release of intracellular contents, and intense inflammatory responses [79]. Oxidative stress, through excessive ROS production, activates RIPK1 autophosphorylation at serine residue 161 and recruits RIPK3 to form necrosomes [80]. Necroptosis synergistically exacerbates tissue injury alongside mitochondrial-dependent apoptosis and ferroptosis.

It is important to emphasize that apoptosis, ferroptosis, and necroptosis are not isolated events, but rather involve complex ‘crosstalk’ against the backdrop of oxidative stress [81]. For example, lipid peroxidation products accumulated during ferroptosis, such as 4-hydroxynonenal (4-HNE), can serve as signaling molecules that further activate pro-inflammatory pathways like NF-κB and sensitize cells to undergo necroptosis [82]. Conversely, the initiation of mitochondrial-dependent apoptosis is accompanied by the collapse of mitochondrial membrane potential and the release of cytochrome c. This process exacerbates ROS production and may release iron ions, thereby creating a microenvironment that favors the occurrence of ferroptosis [83]. The rupture of the cell membrane and release of contents caused by necroptosis trigger a strong inflammatory response, recruiting more immune cells to produce ROS, thereby forming a positive feedback loop that amplifies tissue damage. Ultimately, regardless of the unique entry routes and initial ROS sources of different biotoxins, this initial ROS burst uniformly dictates liver injury by activating this intertwined signaling and cell death network.

## 6. Hepatoprotective Strategies Targeting Oxidative Stress: Clinical Perspectives and Translational Challenges

The liver, owing to its dual vascular supply via the hepatic portal system and metabolic characteristics, is highly susceptible to biotoxin-induced injury [84]. The prevention and treatment of hepatic injury are not merely medical concerns, directly impacting individual health and quality of life, but also exert profound effects on public health security, economic development, and social stability [85]. Therefore, in-depth investigation into the molecular mechanisms of biotoxin-induced hepatic injury and the development of effective preventive and therapeutic strategies are crucial not only for safeguarding individual health but also for maintaining public health security and promoting economic development and social stability.

Toxins directly induce ROS and lipid peroxidation (LPO) production [86]. Concurrently, iron overload exacerbates ferroptosis in hepatocytes [87], while depletion or impaired synthesis of antioxidants leads to hepatic injury [88]. Preventing biotoxin-induced hepatic injury requires comprehensive interventions across multiple levels, including reducing toxin exposure, enhancing hepatic detoxification capacity, blocking toxic pathways, and protecting hepatocyte function, as systematically categorized and evaluated in Table 2.

### 6.1. Direct Antioxidants

Direct supplementation with exogenous antioxidants constitutes a primary strategy to neutralize toxin-induced excessive ROS. For instance, N-acetylcysteine (NAC), as a GSH precursor, is employed to treat acute hepatic injury induced by acetaminophen (APAP) overdose. Its protective effect arises from replenishing GSH reserves and neutralizing the toxic metabolite *N*-acetyl-*p*-benzoquinone imine (NAPQI) [89]. Vitamin E [90] and melatonin [24], among others, mitigate oxidative damage induced by toxins such as MC-LR by scavenging lipid peroxyl radicals [90].

However, such agents lack organelle specificity, making it difficult to effectively concentrate them in primary ROS-producing sites such as mitochondria. While NAC is currently utilized in clinical settings for specific toxicities, for broad biotoxin exposure, issues including low bioavailability, rapid metabolism, and the need for repeated dosing limit their clinical application.

### 6.2. Nrf2 Pathway Activators

Activation of the Nrf2/ARE signaling pathway synergistically upregulates multiple phase II detoxification enzymes and antioxidant genes (such as NQO1, HO-1 and GCL), enhancing the body’s detoxification capacity against toxins like MC-LR and aflatoxins. Small-molecule compounds such as sulforaphane and CDDO-Me have demonstrated significant reduction in hepatic injury in preclinical studies through Nrf2 pathway activation [91]. Despite these promising results, the clinical translation of Nrf2 activators presents distinct challenges.

It is noteworthy that sustained and non-selective activation of Nrf2 may promote unintended cellular proliferation, carrying potential carcinogenic risks over long-term use. Furthermore, during advanced stages of hepatic injury, dysregulation of the Keap1-Nrf2 pathway may diminish activation efficiency.

### 6.3. Ferroptosis Inhibitors

Ferroptosis plays a pivotal role in hepatic injury induced by various toxins (such as APAP, ethanol and MC-LR). Deferoxamine (DFO) and the ferroptosis inhibitor Ferrostatin-1 effectively chelate free iron or scavenge lipid radicals, thereby inhibiting the Fenton reaction and lipid peroxidation chain reactions to mitigate hepatocyte damage [92].

However, existing ferroptosis inhibitors often lack tissue specificity, and systemic iron chelation may induce adverse effects such as anemia and trace element metabolic disorders. Furthermore, these compounds exhibit poor pharmacokinetic properties, with clinical translation remaining in its infancy.

### 6.4. Mitochondria-Targeted Therapies

Mitochondria-targeted antioxidants (MitoQ) can effectively mitigate mitochondrial oxidative stress and improve energy metabolism in models of drug-induced liver injury (such as APAP overdose) [38]. Given that the core toxicological mechanism of MC-LR similarly involves severe mitochondrial electron leakage and structural damage, MitoQ demonstrates immense translational potential in combating such mitochondria-targeting biotoxins.

However, the synthesis of such drugs involves complex processes and high costs, with targeted delivery efficiency and in vivo stability requiring further enhancement. Moreover, mitochondrial function is highly dependent on cell type and pathological environment, necessitating vigilance against metabolic maladaptation resulting from excessive intervention [94]. Ongoing clinical trials are required to validate the safety profile of these targeted therapies in human cohorts.

### 6.5. Combining CRISPR-Mediated Gene Editing with Nanodelivery

CRISPR/Cas9 technology enables direct editing of key oxidative stress genes, such as knocking down Keap1 to sustain sustained activation of Nrf2 signaling, or editing ferroptosis-related genes to alleviate lipid peroxidation [95]. However, safe and efficient in vivo delivery remains a challenge for gene editing therapies. Viral and non-viral nanocarriers (such as liposomal nanoparticles, gold nanoclusters and polymeric complexes) enable liver-specific delivery of sgRNA/Cas9 ribonucleoproteins or exogenous DNA, significantly enhancing editing efficiency while reducing immunogenicity, thereby paving the way for clinical translation [93].

## 7. Gut–Liver Axis: A Key Pathogenic Amplification Pathway for Biotoxin-Induced Liver Injury

In addition to direct hepatotoxicity, the gut–liver axis has emerged as an important physiological regulatory system that can amplify the progression of liver injury induced by various biotoxins, such as AFB1 and MC-LR. The gut–liver axis is a bidirectional information exchange network between the intestinal lumen and the liver, primarily mediated via the portal vein [96]. A growing body of research has confirmed that exposure to biotoxins from environmental and dietary sources can severely disrupt intestinal barrier integrity and alter gut microbiota composition, thereby leading to increased intestinal permeability—a condition clinically referred to as “leaky gut” [97].

After intestinal barrier disruption, pathogen-associated molecules derived from the gut (particularly LPS) and undegraded toxins can undergo gut-derived translocation and enter the portal circulation. Upon reaching the liver, these translocated substances (especially LPS) activate hepatic resident macrophages (Kupffer cells) via Toll-like receptor 4 (TLR4) [98]. This action initiates and amplifies downstream inflammatory cascades, such as the NF-κB signaling pathway, while also exacerbating oxidative stress injury in the body [99].

The interaction between the liver and the intestine forms a vicious cycle: primary liver injury caused by biotoxins impairs liver function, which subsequently compromises intestinal barrier integrity; meanwhile, intestinal endotoxins continuously enter the bloodstream, further exacerbating hepatic inflammation and hepatocyte damage. The synergistic effect of primary biotoxin-induced injury and secondary gut-derived injury significantly accelerates the progression of liver pathology, ultimately leading to liver fibrosis, steatohepatitis, and even hepatocellular carcinoma [100].

Therefore, therapeutic strategies for hepatotoxicity induced by biological toxins should not be limited to local antioxidant and anti-inflammatory interventions in the liver. Preserving intestinal barrier function and modulating gut microbiota composition to reduce endotoxin translocation represent a highly promising adjunctive therapeutic direction for interrupting the pathogenic cascade of the gut–liver axis at a systemic level.

## 8. Conclusions

In summary, this review establishes oxidative stress as the central, convergent mechanism underlying diverse biotoxin-induced hepatic injuries. By elucidating these shared molecular pathways, we provide a comprehensive logical framework that reveals the intrinsic patterns of disease progression and offers a theoretical foundation for subsequent research.

Understanding these mechanisms is critical for developing precise hepatoprotective strategies. While traditional antioxidant agents, such as GSH, have demonstrated potential by scavenging excess free radicals [101], emerging evidence highlights the efficacy of specific modulators—including black tea extract [102], astaxanthin (ASX) [103], and cholic acid (CA) [104]—in enhancing hepatic detoxification and anti-inflammatory responses. Furthermore, drugs targeting cellular defense mechanisms, such as the Nrf2 signaling pathway, are gaining prominence. Consequently, future therapeutic strategies must evolve from broad-spectrum antioxidants to targeted redox interventions, including mitochondria-directed therapies and specific cell death inhibitors.

Despite these therapeutic advances, clinical management faces significant real-world challenges, primarily due to the complexity of environmental exposures. Biotoxins frequently coexist with chemical pollutants (heavy metals and microplastics) during food production, storage, and processing. This mixed exposure, exacerbated by global environmental changes, poses a severe and synergistic threat to liver health. For instance, AFB1 exposure significantly increases the risk of hepatocellular carcinoma when combined with hepatitis C infection or alcohol consumption [105]. Similarly, environmental microcystin potentiates liver damage induced by the hepatitis B virus and aflatoxins [106]. However, current research remains largely confined to single-toxin models, leaving a critical gap in our understanding of synergistic toxicity mechanisms.

Furthermore, susceptibility to biotoxin-induced injury is highly dependent on individual variations, such as genetic predispositions, lifestyle habits, and the gut microbiota. Substantial evidence implicates the gut–liver axis in disease progression; biotoxins can alter ileal microbiota and facilitate the translocation of enteric LPS via the portal venous circulation, thereby exacerbating hepatic inflammation [17].

To address these escalating environmental health threats, future research must prioritize the establishment of robust mixed-exposure in vivo and in vitro models. By deeply exploring synergistic molecular mechanisms and integrating individual variability factors like microbiome-host interactions, the field can transition toward developing personalized, highly specific preventive measures and targeted antidotes, ultimately ensuring global liver health.

## Figures and Tables

**Table 1:** Comparative Hepatotoxicity Phenotypes, ROS Sources, and Mechanisms Induced by Diverse Biotoxins.

Toxin Category	Representative Toxins	Primary ROS Source	Hepatotoxic Phenotype	Major Signaling Pathways & Cell Death	Potential Therapeutic Targets	Reference
Mycotoxins	AFB1	CYP450 catalytic cycle	Hepatocyte necrosis, cholangiocyte hyperplasia, hepatic steatosis and hepatic hemorrhage	DNA adduct formation, apoptosis, necrosis	Nrf2 pathway activators	[18,20,21,22,23]
	OTA	Mitochondrial dysfunction	Hepatocyte degeneration, necrosis, apoptosis, inflammation, fibrosis	Mitochondrial pathway apoptosis	Direct antioxidants, mitochondria-targeted therapies	[24,25,26]
Phytotoxins	PAs	CYP450 activation, Mitochondria	Severe hepatic sinusoidal congestion, fibrotic occlusion of hepatic venules	Nrf2/ARE compensatory activation, necrosis	Antioxidants, CYP450 modulators	[27,28,29,30]
	Ricin	Mitochondria (GSH depletion)	Diffuse hepatocyte necrosis	Ferroptosis (iron overload, GPX4 inhibition)	Ferroptosis inhibitors	[31,32]
Bacterial/Cyanobacterial toxins	LPS	Macrophages, NADPH Oxidase (NOX)	Neutrophil infiltration, hepatocyte punctate necrosis, maintenance and exacerbation of steatohepatitis	TLR4/NF-κB inflammatory cascade	NOX inhibitors, anti-inflammatory agents	[33,34]
	MCs	Mitochondrial ETC disruption	Hepatocyte sloughing, hepatic fibrosis, hepatocellular carcinoma	Apoptosis (Bax/Caspase-3), PP1/PP2A inhibition	Mitochondria-targeted antioxidants	[35,36,37,38]

**Table 2:** Hepatoprotective strategies targeting oxidative stress.

Intervention Type	Representative Drug/Technique.	Advantages	Disadvantages	Reference
Direct antioxidant	N-acetylcysteine (NAC), Vitamin E, melatonin	Acts in a direct manner and can effectively clear ROS, antagonizing liver injury induced by toxins.	Lacking organelle targeting, it is difficult to enrich in mitochondria and other major ROS generation sites; low bioavailability and rapid in vivo metabolism require repeated administration, limiting its clinical application.	[24,89,90]
Nrf2 pathway activator	Sulforaphane, CDDO-Me	It can enhance antioxidant and detoxification capabilities at the endogenous level, and animal experiments have confirmed that it significantly alleviates toxin-induced liver injury.	Persistent activation of Nrf2 poses a potential carcinogenic risk; dysregulation of pathway feedback in late-stage liver injury leads to reduced activation efficacy.	[63,91]
Ferroptosis inhibitor	DFO, Ferrostatin-1	Can specifically inhibit ferroptosis, effectively alleviating hepatocyte injury induced by various toxins.	Lack of tissue specificity; systemic administration tends to cause side effects such as anemia and trace element disorders; poor pharmacokinetic performance; immature clinical translation	[32,92]
Mitochondria-targeted therapy	MitoQ	Precisely targets mitochondrial ROS generation. Although its prominent hepatoprotective effects are mainly validated in drug-induced models (APAP), it holds significant translational potential for combating mitochondria-targeting biotoxins (MC-LR and OTA).	Drug synthesis is difficult and costly; targeted delivery efficiency and in vivo stability require optimization; it is prone to causing abnormal cellular metabolism.	[38]
CRISPR gene editing combined with nanodelivery technology.	Editing oxidative stress and ferroptosis-related genes using CRISPR/Cas9; combined with nanocarriers such as LNPs and gold nanoclusters for liver-specific delivery to enhance editing efficiency and reduce immunogenicity.	It can achieve long-term regulation of pathological pathways at the genetic level; nanocarriers enable liver-targeted delivery with high editing efficiency and low immunogenicity.	In vivo safe and efficient delivery remains a core challenge, and the overall technology has yet to complete clinical translation.	[93]

## Data Availability

No new data were created or analyzed in this study. Data sharing is not applicable to this article.
